# The Wrist Injury That Never Got Better

**Published:** 2016-05-24

**Authors:** Saptarshi Biswas, Molly Philbin

**Affiliations:** Department of Trauma and Acute Care Surgery, Forbes Regional Hospital, Allegheny Health Network, Pittsburgh, PA

**Keywords:** arthritis, wrist, scaphoid nonunion, arthrodesis, scapholunate advanced collapse (SLAC)

**Figure:**
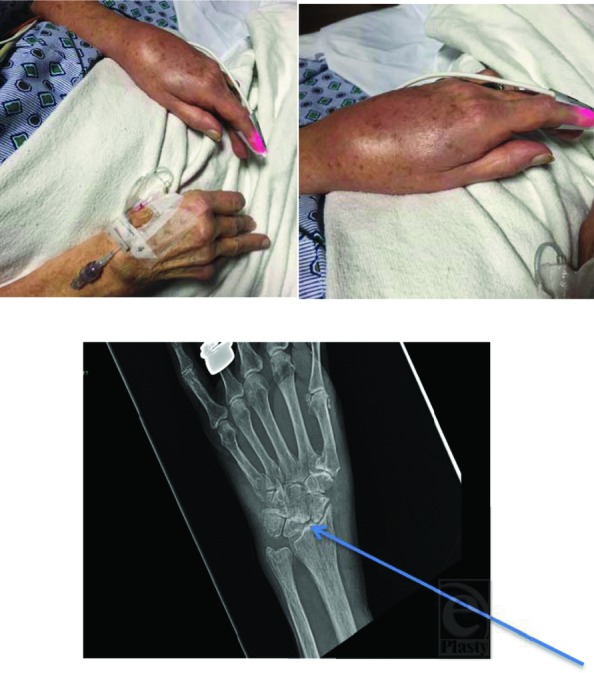


## DESCRIPTION

An 85-year-old woman involved in a motor vehicle accident presented with an incidental finding of swollen, painful left wrist that started insidiously after a fall 6 years back and progressively getting worse.

## QUESTIONS

1. **What is the diagnosis?**
2. **What are the common etiologies?**
3. **What is the underlying pathogenesis?**
4. **What are the treatment options?**

## DISCUSSION

This is a case of scaphoid lunate advanced collapse (SLAC), where progressive instability results in advanced arthritis of the radiocarpal and midcarpal joints.^1^

SLAC wrist develops following weakness of the scapholunate ligament. It can be traumatic secondary to a scaphoid fracture that has resulted in nonunion.^2^ Atraumatic causes include calcium pyrophosphate dehydrate deposition disease, rheumatoid arthritis, neuropathic diseases, and β_2_-microglobulin-associated amyloid deposition disease.^3^^,^^4^

The scapholunate ligament binds and holds the scaphoid and lunate in place. Chronic injury to this ligament can lead to an extension of the lunate and subsequent flexion of the scaphoid, which causes an irregular distribution of the forces in the joints starting with the radioscaphoid joint. The severity of SLAC is based on Watson criteria.^1^

Watson stages are as follows:

- *Stage I:* Arthritis between scaphoid and radial styloid.
- *Stage II*: Arthritis between scaphoid and entire scaphoid facet of the radius.
- *Stage III*: Arthritis between capitate and lunate.

Some physicians include a stage IV describing a pancarpal arthritis including the radiolunate joint that is normally spared.

Asymptomatic (SLAC) wrist generally does not require treatment. Symptomatic mild SLAC is managed nonoperatively, for example, wrist immobilization with splints, nonsteroidal anti-inflammatory drugs, and corticosteroid injections.^2^ Symptomatic SLAC, refractory to conservative management, requires operative intervention, which is planned on the basis of radiographic findings, stage of arthrosis, symptoms, physical examination findings, and the surgeon's preference.

1. *Wrist denervation*: Used by itself or in combination with other surgical procedures is effective in the setting of chronic wrist pain. The terminal branches of anterior interosseous nerve and the posterior interosseous nerve are usually responsible for painful stimuli at the wrist.^5^^,^^6^ The denervation may be complete or incomplete. Incomplete posterior interosseous nerve neurectomy is commonly performed.^2^
2. *Radial styloidectomy*: Early symptomatic arthrosis in SLAC wrist presents between the radial styloid and the scaphoid and hence radial styloidectomy can be of utility in cases of more advanced disease. With radiocarpal abutment, it can be used in combination with other surgical procedures.^2^
3. *Partial wrist arthrodesis*: Four-corner arthrodesis is usually preferred in active, young patients, especially when capitolunate arthritis is present.^2^
  I. Four-corner arthrodesis—with K-wires and screws
  II. Four-corner arthrodesis—with circular plates. Developed as an alternative to the former, with arguably relatively stable fixation and lower nonunion rate. Outcomes have been far from satisfactory.
  III. Capitolunate arthrodesis: Advantages include easier lunate reduction postexcision of the triquetrum, as well as avoiding symptomatic pisotriquetral arthritis. Recent studies have underlined the benefits of capitolunate arthrodesis having similar outcomes to standard 4-corner arthrodesis.
4. *Proximal row carpectomy*: First described by T. T. Stamm is an established operative treatment of radiocarpal arthrosis.^7^ Technically easy with lack of implanted fixation, it often allows better preservation of strength and motion (compared with limited carpal arthrodesis). Patients expect 60% of normal range of motion compared with contralateral wrist and more than 90% of normal grip strength in comparison with 4-corner fusion where patients expect less than 50% range of motion and approximately 75% grip strength.^8^ However, capitolunate arthrosis and advanced carpal collapse are relative contraindications.
5. *Total wrist arthrodesis*: The ultimate salvage procedure for any motion preservation. The conversion rates of both 4-corner arthrodesis and proximal row carpectomy to total wrist arthrodesis are approximately 5%.^2^
